# Effects of Storage Temperature on the Microbial Flora, Odor, and Quality of Shucked Pacific Oysters Under Mimicked Commercial Shipping Conditions

**DOI:** 10.3390/foods15040603

**Published:** 2026-02-07

**Authors:** Nao Watakabe, Kazuho Sakaguchi, Mikiko Tomatsu, Akane Matsumoto, Ayumi Furuta, Takashi Okazaki, Shota Tanimoto

**Affiliations:** 1 The Graduate School of Comprehensive Scientific Research, Prefectural University of Hiroshima, Hiroshima 734-8558, Japan; n-watakabe24043@pu-hiroshima.ac.jp (N.W.); r431002hv@ed.pu-hiroshima.ac.jp (M.T.); 2 Faculty of Regional Development, Prefectural University of Hiroshima, Hiroshima 734-8558, Japan; m251482@hiroshima-u.ac.jp (K.S.); amatsumoto@pu-hiroshima.ac.jp (A.M.);; 3 Department of Food and Nutrition, Sanyo Women’s College, Hatsukaichi 738-8504, Japan; okazaki@sanyo.ac.jp

**Keywords:** refrigerated temperature, microbiota, next generation sequencing, volatile organic compounds, storage, shelf-life extension

## Abstract

The occurrence of unpleasant odors during the storage of shucked oysters shipped in brine is considered a critical issue in the improvement of oyster quality. This study focused on the storage temperature as a strategy to control odor development and assessed storage conditions at different temperatures. Analyses focused on the bacteria (total viable count and bacterial flora), biochemical induces (pH and total volatile basic nitrogen), and odor (sensory evaluation and volatile organic compounds) of oyster meat and brine. In sensory evaluation and odor sensor measurements, odor intensity remained unchanged at 0 °C but increased significantly at 3 °C. Odor generation was confirmed to be decreased at low temperatures. In addition, compared to 3 °C storage, 0 °C storage effectively suppressed the increase in viable bacterial count, the production of volatile organic compounds, the production of trimethylamine, and the decrease in pH. In particular, the proliferation of *Psychrilyobacter* and *Psychromonas* and the production of 1-propanol and short-chain fatty acids were significantly inhibited. In oyster meat, aldehyde production was significantly suppressed at 0 °C. Furthermore, total volatile basic nitrogen levels were also lower than those at 3 °C. Therefore, even a slight temperature difference contributes to improved quality, suggesting that temperature management during storage plays a crucial role in quality changes.

## 1. Introduction

Farmed seafood products are typically harvested from aquaculture facilities, transported to processing plants, and subsequently processed, packaged, and distributed [1]. The Pacific oyster (*Magallana gigas*) is one of the most widely farmed shellfish species. Oysters are commonly distributed in the shell or shucked forms; the latter are typically prepared by skilled workers at processing facilities [2]. The retail distribution of shucked oysters offers convenience by eliminating the need for shell removal during cooking. Shucked oysters are more acceptable to consumers than oysters with shells [3]. Despite the high consumer demand for raw shucked oysters, they are highly perishable and have a limited shelf life. In many Asian countries, oysters are sold at retail stores after being shucked, packaged, and shipped, and inadequate hygiene management may pose a risk to human health due to bacterial proliferation [4,5,6]. In addition, for long-distance transportation and broader distribution, maintaining freshness over extended periods is essential. Therefore, the development of an effective storage method to preserve the quality of shucked oysters over a long period of time is of great practical importance.

During the packaging process, the traditional method of packaging shucked oysters in brine has been adopted to preserve the fragile structure of oysters [7,8]. When shucked oysters were stored in brine, dehydrogenase activity in the oyster gills remained even after two days, indicating survival [9]. This storage method can effectively suppress the deterioration of freshness. Furthermore, in Hiroshima Prefecture and Miyagi Prefecture, Japan, renowned oyster-producing regions, standards have been established for brine used to soak shucked oysters, and this storage method is a widely used and common practice [8,10]. However, this preservation method causes the leaching of free amino acids and nucleic acid-related compounds from the adductor muscle [7,11]. Furthermore, an unpleasant odor develops in the brine on the sixth day of refrigerated storage [5,6]. Volatile organic compounds (VOCs), such as short-chain fatty acids and esters, increased significantly in shucked oysters soaked in brine and in the brine [12]. An unpleasant odor was detected in both the brine and oysters, highlighting this as a significant issue during storage.

The odor of shellfish changes depending on the storage conditions in the supply chain, resulting in a decline in the quality of seafood [13]. VOCs produced by microbial metabolism contribute to spoilage and unpleasant odors [14]. The unpleasant odor in shucked oysters is owing to bacterial growth [5,6,12]. We previously reported that bacteria proliferating on shucked oysters soaked in brine differed from those proliferating on intact oysters stored in air [12]. This difference led to differences in the VOC generation levels. However, the mechanisms by which VOC are produced in oysters during storage and the relationship between VOCs and bacterial flora remain unclear.

Storage temperature is a critical factor that influences shellfish quality. Elevated temperatures promote the growth of microbes, including pathogenic species [15]. The growth rate of *Vibrio parahaemolyticus* in oysters varies with storage temperature [16,17], and bacterial growth in brine is inhibited at lower temperatures [6]. Additionally, in clams, the storage temperature affects not only microbial proliferation but also the production of taste- and odor-related compounds. Shelled clams stored at 5 °C and 10 °C exhibited higher levels of malic acid and free amino acids compared to those stored at temperatures higher than 20 °C [18], while trimethylamine (TMA), a compound associated with unpleasant odors, was detected in shelled oysters stored at 20 °C but not at 5 °C [19]. Furthermore, during the storage of HPP-treated shucked oysters, freezing enables longer-term quality maintenance than refrigeration [20]. Therefore, storage temperature plays a critical role in the quality of seafood products, including shellfish, and maintaining lower temperatures over extended storage periods may help preserve oyster quality. Specific standards for the storage temperature have been established for shucked oysters. According to the Ministry of Health, Labour, and Welfare (in Japan) [12,21], raw oysters should be stored at temperatures below 10 °C. Furthermore, the “Guidelines for the Handling of Raw Oysters” issued by Hiroshima Prefecture [10] recommend maintaining refrigerator temperatures of ≤5 °C during storage. However, the effects of storage temperature on VOC generation and changes in the bacterial microbiota during the storage of shucked oysters remain unclear. Clarifying this would reveal the storage conditions necessary to obtain high-quality shucked Pacific oysters.

Therefore, this study aimed to determine the optimal storage temperature for maintaining the quality of shucked Pacific oysters by investigating the effects of storage temperature on quality changes in shucked oysters immersed in brine. In addition to conventional quality indicators (viable bacterial counts, pH, TMA, and total volatile basic nitrogen [TVB-N]) and the sensory characteristics of oyster meat and brine stored at different temperatures, VOCs and microbial communities were also evaluated. Furthermore, the relationships between these factors were examined. These analyses, particularly the assessment of time-series changes in VOCs and microbial communities, provide new insights into the behavior of quality changes in shucked oysters during storage. The findings obtained under realistic storage conditions involving brine immersion offer practical implications for developing strategies to maintain oyster freshness during distribution and marketing.

## 2. Materials and Methods

### 2.1. Sample Preparation and Storage Conditions

Because the distribution volumes of oysters in Japan are high in February, a storage trial was conducted in February 2024 to evaluate the preservation conditions of raw oysters. Fresh oysters (*Magallana gigas*) were obtained from a dedicated aquaculture farm in Hiroshima Bay, Japan. The oysters were shucked by trained personnel at a seafood-processing company in Kure City, Hiroshima Prefecture. The average weight of each shucked oyster was 20.2 ± 3.9 g (mean ± standard deviation).

The storage trial and sample preparation were performed based on previous reports [5,6,7,8,11,12] with some modifications. Five pieces of shucked oyster meat were placed in a polyethylene film container (thickness: 0.04 mm; SEISANNIPPONSHA, Tokyo, Japan), along with an equal quantity of brine (seawater without sterilization collected from the same marine area). The containers were sealed using a heat sealer (SURE Sealer, NL-301P; ISHIZAKI ELECTRIC MFG Co. Ltd., Tokyo, Japan).

Storage conditions were categorized into two temperature settings: (1) 3 °C, the typical temperature inside a standard refrigerator; and (2) 0 °C, the condition for ice storage commonly used for seafood. These temperature settings were selected based on regulatory guidelines [10,12]. For cold storage, samples were placed in temperature-controlled refrigerators set to 3 °C. During the storage trial at 3 °C, we monitored the temperature by a sensor placed into the refrigerators and confirmed that temperature fluctuations remained within ±1 °C For ice storage, samples were placed in polystyrene containers filled with ice, with additional ice replenished every 2 days to maintain the temperature at approximately 0 °C. During storage at 0 °C, the temperature was monitored using a thermometer, and the absence of fluctuations was confirmed. Each sample was stored for 12 days. The sampling days were as follows: fresh (day 0), immediately after placing oyster meat in brine (day 1), just before the expiration date (day 3), past the expiration date (day 7), and notably past the expiration date (day 12). This expiration framework was based on Japanese guidelines [6,7].

To account for individual variation, five oyster meat samples were minced and mixed together to create a sample. The storage trial was conducted with a sample size of three (*n* = 3). All analytical measurements, except for viable bacterial counts, were conducted on samples preserved at −80 °C.

### 2.2. Viable Bacterial Count

For the analysis of viable bacteria, 10 g of oyster meat was weighed and homogenized in 90 mL of sterile physiological saline (0.85% NaCl) for 2 min using a stomacher (Masticator Homogenizer, IUL, Barcelona, Spain). The brine samples were used without pretreatment. Both samples were serially diluted with sterile physiological saline, and viable bacterial counts were determined using the plate count method.

Four types of culture media were used to quantify different bacterial groups: Standard Plate Count Agar for mesophilic bacteria, Marine Agar for heterotrophic marine bacteria, Salt-Enhanced Bonito-peptone-glucose Agar for psychrophilic bacteria, and thiosulfate citrate bile sucrose agar for *Vibrio parahaemolyticus*. The composition of each medium and incubation conditions were consistent with those reported in our previous study [12]. After incubation, colony-forming units (CFU) were counted, and the results were expressed as CFU per gram of sample. All values were converted to a logarithmic scale (log CFU/g).

### 2.3. Microbial Flora Analysis

Bacterial community analysis was performed on oyster meat and accompanying brine samples obtained at three time points: immediately after packaging (day 0), prior to the expiration date (day 3 at 0 °C and 3 °C), and significantly beyond the expiration date (day 12 at 0 °C and 3 °C).

DNA extraction and polymerase chain reaction (PCR) amplification were conducted according to previously reported protocols [12], with minor modifications to the analytical procedures to suit the experimental conditions of the present study. Briefly, a 10 g portion of oyster meat was homogenized with 90 mL of sterile physiological saline (0.85% NaCl) and centrifuged to obtain a suspension.

Genomic DNA was extracted from the suspension using the ZymoBIOMICS™ DNA Miniprep Kit (Zymo Research Corporation, Irvine, CA, USA), following the manufacturer’s protocol. The V3–V4 region of the 16S rRNA gene was amplified via PCR and the resulting amplicons were verified using electrophoresis on a 2% agarose gel. Index PCR was subsequently performed, and sequencing was conducted using the NextSeq 1000 and MiSeq platforms (Illumina, San Diego, CA, USA) under paired-end 2 × 300 bp conditions.

Post-sequencing and metagenomic analyses were performed using **Qiime2** (https://qiime2.org/, accessed 17 November 2025). These included amplicon sequence variant (ASV) clustering, the calculation of alpha diversity indices, and principal coordinate analysis (PCoA) based on beta diversity. During data preprocessing, sequences assigned to cyanobacteria, mitochondria, eukaryota, unclassified animals, chloroplast, and *Mycoplasma* were excluded. Dominance and alpha diversity metrics were calculated based on the filtered dataset. PCoA based on weighted UniFrac distances was performed to visualize the differences in the microbial communities. The analysis was conducted using the R software (version 4.5.1, https://www.r-project.org/, accessed on 17 November 2025).

### 2.4. Determination of pH, TVB-N, and TMA

To measure pH, oyster meat (1.0 g) was homogenized with 5 mL of distilled water. The pH values of the homogenates were measured using a pH meter.

TVB-N and TMA concentrations were determined according to a method described previously [12]. Briefly, for both measurements, 1.0 g of sample was extracted with 10 mL of 5% trichloroacetic acid. TVB-N was quantified using Conway’s microdiffusion method and TMA was measured using gas chromatography–mass spectrometry (GC-MS).

### 2.5. Sensory Evaluation and Odor Sensor Analysis

Sensory evaluation was conducted by 34 panelists (mean age: 26.6 ± 6.9 years) recruited from the Prefectural University of Hiroshima. The evaluation focused on the odor intensity of oyster meat and brine, the intensity of spoilt odor, and the overall odor pleasantness of oyster meat. A 5-point hedonic scale was used, where 1 indicated “very weak or very unpalatable” and 5 indicated “very strong or very palatable,” as shown in Table 1.

For the spoilage odor intensity of oyster meat, reference samples were defined as follows: day 0 oyster meat was assigned a score of 1, and oyster meat stored at 3 °C for 12 days was assigned a score of 5. For brine odor intensity, day 0 brine (seawater) was used as the reference with a score of 1. No reference criteria were set for the general odor intensity of oyster meat, and the panelists were instructed to evaluate odors based on their subjective perception. The evaluation criteria were established through a preliminary trial involving a small number of participants. Panelists have been trained in advance using reference samples. The sensory evaluation protocol involving human subjects was approved by the Research Ethics Committee of the Prefectural University of Hiroshima (approval number 23HH009).

Odor intensity was also measured using an odor sensor (XP-329IIIR; Shin Cosmos Electric Co., Ltd., Tokyo, Japan). The measurements were conducted in batch mode, with the peak value recorded at the time of sample opening. For all evaluations, 5.0 g of the sample was sealed in a 25 mL vial, and the odor intensity was measured immediately upon opening the vial.

### 2.6. Identification and Quantification of VOCs

VOCs were extracted using headspace solid-phase microextraction and analyzed using GC-MS (Shimadzu Corporation, Kyoto, Japan). The analytical procedure was based on the method described by the preceding report [12,19,22], with minor modifications. Briefly, 1.4 g of oyster meat was homogenized in 7 mL of saturated saline solution and centrifuged at 12,000× *g* for 10 min. Five milliliters of the supernatant was collected. The brine samples were supplemented with 2.0 g of sodium chloride to saturate the solution and thereby enhance the extraction of VOCs. Microextraction was incubated at 40 °C for 60 min and absorbed onto a 50/30 μm DVB/CAR/PDMS fiber (fiber length: 2 cm) for 30 min.

GC-MS analysis was performed in split-less mode. For samples in which peak intensities exceeded the detectable range in split-less mode, additional analyses were performed using the split mode (split ratio = 10.0). The concentration of each VOC was calculated based on the peak area obtained; then, principal component analysis was performed using SIMCA 16.0 to create score plots and loading plots.

### 2.7. Statistical Analysis

Statistical analyses were performed using SPSS Statistics software (version 29.0.2.0; IBM Corp., Armonk, NY, USA). Statistical significance was set at *p* < 0.05 for all tests. For comparisons involving three or more groups, a one-way analysis of variance (ANOVA) was followed by Tukey’s multiple comparison test. For comparisons between two groups, either Student’s *t*-test or Welch’s *t*-test was applied, depending on the equality of variances. Correlation analysis was performed in SPSS Statistics (version 29.0.2.0; IBM Corp., Armonk, NY, USA) to investigate the correlation between bacterial genera and VOCs using Pearson’s correlation coefficient. Based on these results, a heatmap was generated using Google Colab (https://colab.research.google.com/; accessed on 12 October 2025). For each measurement item (pH, TVB-N, TMA, and odor intensity using an odor sensor), analyses were performed in triplicate on the same sample, and the results are expressed as the mean ± standard deviation.

## 3. Results and Discussion

### 3.1. Viable Bacterial Counts

**Figure 1:**
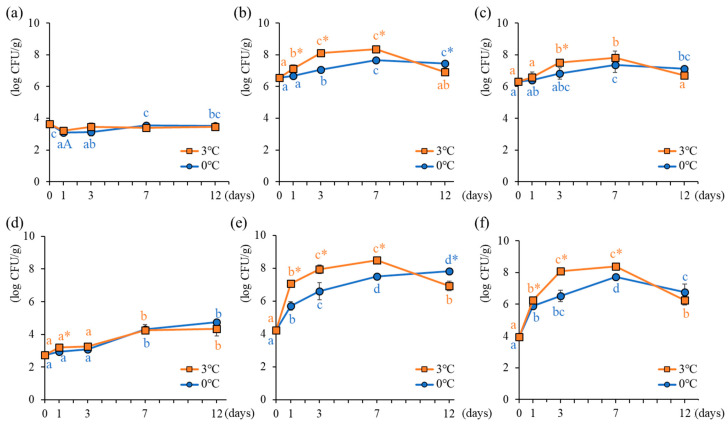
Time-dependent changes in viable bacterial counts in oyster meat and brine stored at different temperatures. (**a**–**c**) viable bacteria counts in oyster meat, (**d**–**f**) viable bacteria counts in brine. (**a**,**d**) mesophilic bacterial counts, (**b**,**e**) heterotrophic marine bacterial counts, (**c**,**f**) psychrophilic bacterial counts. Different markers show the mean values of different temperature conditions, while the bars indicate standard deviation at each storage time. Different lowercase letters indicate significant changes (*p* < 0.05) during storage time. * indicates significant variations in storage temperature (*p* < 0.05).

Figure 1 illustrates changes in viable bacterial counts in the oyster meat and brine samples during storage. *Vibrio parahaemolyticus* was not detected in any of the samples. This finding is consistent with a report indicating that *V. parahaemolyticus* does proliferate at temperatures below 10 °C [17]. It can be inferred that both conditions established in this study were suitable for inhibiting the growth of *V. parahaemolyticus* in oysters.

In oyster meat, mesophilic bacterial counts remained relatively stable across all storage conditions, ranging from 3.08 to 3.64 log CFU/g (Figure 1a), and were approximately three orders of magnitude lower than those observed in other media. In Japan, viable bacterial counts measured using standard agar medium serve as indicators to assess the quality of raw oysters [10]. However, viable bacterial counts did not increase significantly in this medium; nevertheless, an unpleasant odor developed at 3 °C, confirming quality deterioration (as described Section 3.4). In contrast, heterotrophic marine bacteria and psychrophilic bacteria exhibited significant changes depending on storage temperature and duration (Figure 1b,c). Heterotrophic marine bacteria showed the highest counts, with significant increases observed until day 12 at 0 °C and day 7 at 3 °C, followed by a decline at 3 °C. The decrease observed on the 12th day of storage at 3 °C was likely due to the bacterial growth progressing, reaching a stationary phase, and then shifting into the death phase [23]. Differences in the storage temperature resulted in changes in the time at which the maximum peak was observed. Notably, on day 3, bacterial counts at 0 °C were significantly lower by approximately one order of magnitude than those at 3 °C, indicating that lower temperatures delayed the proliferation of heterotrophic marine bacteria and psychrophilic bacteria. During the refrigerated storage of shell-on oysters in a supply chain context, low-temperature management suppresses *Escherichia coli* and *Vibrio parahaemolyticus* growth [24]. Additionally, the inhibition of bacterial growth during ice storage has also been reported in shucked oysters (without soaking in brine) [25], Pacific white shrimp [26], and yellowtail [27]. Shucked oysters soaked in brine similarly exhibited temperature-dependent inhibitory effects on bacterial growth.

These changes in viable bacterial counts were more pronounced in brine samples than in oyster meat samples. Unlike oyster meat, mesophilic bacterial counts in brine increased during storage, although differences were not observed between 0 °C and 3 °C storages (Figure 1d). For heterotrophic marine bacteria, counts at 0 °C were significantly lower than those at 3 °C from days 1 to 7 (Figure 1e). However, by day 12, the bacterial counts at 0 °C exceeded those at 3 °C. Nagai et al. [5] reported that when sterilized seawater was used for soaking shucked oysters, bacterial counts in brine stored at 0 °C did not increase until the sixth day of storage, unlike storage at 4 °C and 10 °C. This finding is consistent with the results of this study. The results of the brine samples exhibited bacterial changes similar to those of the oyster meat samples, suggesting that bacterial changes occurred similarly in both. Furthermore, the viable bacterial count in brine differs by up to two orders of magnitude between 0 °C and 3 °C. Psychrophilic bacteria exhibited growth inhibition owing to low temperatures from day 1, and this difference emerged earlier in the brine than in the oyster meat (Figure 1f). Therefore, it was clarified that inhibition of bacterial growth at 0 °C was more pronounced in brine than in oyster meat.

### 3.2. Bacterial Community Structure

Table 2 presents the α diversity indices and ASV counts, which reflect species richness and evenness within the microbial community. All samples exhibited Good’s coverage values exceeding 0.99, confirming the reliability of the sequencing data.

In oyster meat, no significant changes in α diversity and ASV counts were observed during storage, and slight variations in the storage temperature did not substantially affect microbial diversity. In contrast, the brine samples exhibited a marked decrease in diversity over time. Initially, on day 0, brine (freshly collected seawater) exhibited higher diversity than oyster meat, but this difference diminished as storage progressed. At 3 °C, a significant reduction in all diversity indices was observed by day 3, whereas at 0 °C, only the Shannon index showed a significant decline. Overall, higher diversity was maintained in brine stored at 0 °C than that stored at 3 °C, suggesting that the storage at lower temperatures mitigated microbial community shifts.

**Figure 2:**
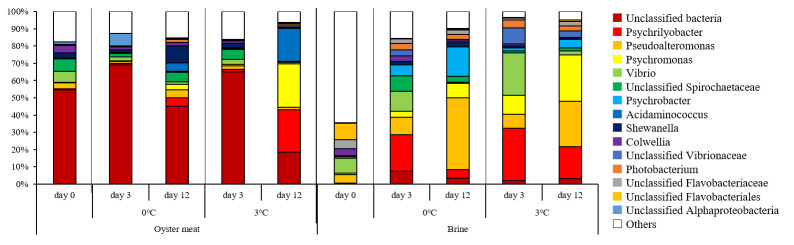
Relative abundance of bacterial genera in oyster meat and brine stored at different temperatures.

Figure 2 shows the genus level composition. In oyster meat on day 0, the dominant genera included unclassified Spirochaetaceae (7.1%), *Vibrio* (6.5%), *Colwellia* (4.3%), and *Pseudoalteromonas* (3.5%), which is consistent with our previous findings [12]. Before storage in the brine, unclassified Flavobacteriales (9.5%), *Vibrio* (8.8%), and *Candidatus Actinomarina* (7.1%, including “Others”) were dominant; however, none exceeded 10% relative abundance, indicating a diverse microbial community. Furthermore, the most abundant unclassified Flavobacteriales and *Candidatus Actinomarinidae* were completely absent in the oyster meat, confirming that the microbial community differs from that found in oyster meat.

Storage temperature influenced bacterial-community dynamics. At 3 °C, oyster meat samples on day 12 were dominated by *Psychromonas* (25.2%), *Psychrilyobacter* (24.7%), and *Acidaminococcus* (19.1%). This change in oyster meat bacterial flora is consistent with the results of our previous study [12]. At 0 °C, *Shewanella* (9.7%) was most abundant, followed by unclassified Spirochaetaceae (5.6%) and *Acidaminococcus* (5.0%), suggesting suppressed proliferation of *Psychrilyobacter* and *Psychromonas* under ice storage (3.3% and 5.0%, respectively, on day 12 at 0 °C). *Psychromonas*, abundant in marine sediments, is an obligate psychrophile (optimal growth temperature 15 °C), which proliferates in the presence of 3–5% salinity [28,29]. *Psychrilyobacter* is known to be an intestinal bacterium of shellfish and has been detected in oyster flesh [5,12,30]. These two genera include psychrophilic bacteria characterized by their ability to survive in high-salinity environments, suggesting that they proliferated under the conditions used in this study. However, the failure of the bacteria to proliferate at 0 °C compared to storage at 3 °C suggests that ice storage may be suitable for suppressing their proliferation.

**Figure 3:**
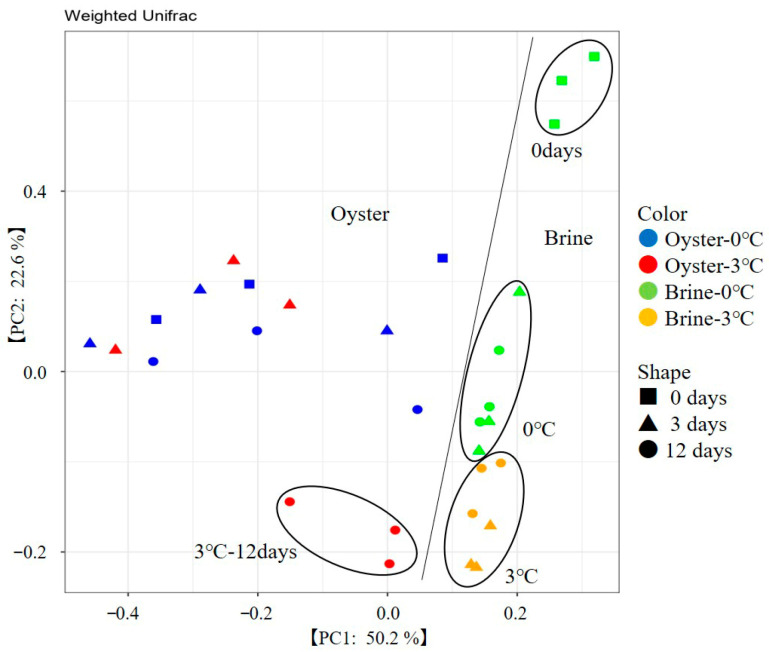
Principal coordinate analysis of bacterial composition (genus level) in oyster meat and brine stored at different temperatures.

On the 12th day, in brine samples at 0 °C, *Pseudoalteromonas* was the most dominant genus, accounting for 41.6% of the bacterial community, followed by *Psychrobacter* (17.3%). In contrast, on the 12th day at 3 °C, *Psychrilyobacter*, *Psychromonas*, and *Pseudoalteromonas* were present at comparable proportions (18.8%, 26.9%, and 26.2%, respectively). As previously reported [12], the growth of *Pseudoalteromonas* and *Psychrobacter*, which did not increase during the storage of oyster samples, was confirmed in the brine samples. *Pseudoalteromonas* and *Psychrobacter* exhibited higher relative abundance at 0 °C than at 3 °C (41.6% and 17.3%, respectively, on day 12 at 0 °C). On the other hand, at 3 °C, the proportion of *Psychrilyobacter* and *Psychromonas* were present at a higher proportion than at 0 °C. The difference in proportions due to storage temperature was presumed to be influenced by the increase in *Psychrilyobacter* and *Psychromonas*. These results are thought to correspond with the lower viable bacterial counts in oyster meat and brine during the storage at 0 °C shown in Figure 1, particularly the significant suppression observed in the brine. Furthermore, the decrease in bacterial diversity during storage was more pronounced in the brine solution than in oyster meat. This may be attributed to the brine solution providing a more favorable environment, such as nutrient conditions, for the proliferation of specific bacteria, potentially leading to their dominance and a consequent reduction in overall bacterial diversity compared to that in oyster meat. However, further research is required to substantiate this hypothesis (Figure 3). These results indicated that specific bacteria (*Psychrilyobacter* and *Psychromonas*) were suppressed from proliferating during low-temperature storage.

PCoA results are shown in Figure 3. The first principal component (50.2%) clearly distinguished oyster meat samples from brine samples. Among the oyster meat samples, the 3 °C storage samples on day 12 was positioned most negatively on the second principal component (22.6%) axis, while the other samples were positioned differently from them. This observation may be owing to the increased proportion of *Psychromonas* and *Psychrilyobacter*, which were highly dominant at 3 °C on day 12. For the brine samples, the samples on day 0 were at the most positive position on the vertical axis and shifted significantly toward the negative position during storage. Furthermore, the samples stored at 3 °C were more negatively positioned than those stored at 0 °C, clearly differentiated by storage temperature. This aligns with the observation in brine samples that significant differences in viable bacterial counts owing to temperature were evident after 3 days of storage (Figure 1). Additionally, the difference observed on the third day in the brine samples did not occur in the oyster meat samples, suggesting that the brine undergoes quality changes before the oyster meat. Based on these results, it was inferred that the first principal component distinguished between the bacterial communities in the oyster meat and brine, whereas the second principal component indicated changes in each bacterial community.

### 3.3. pH, TMA, and TVB-N

**Figure 4:**
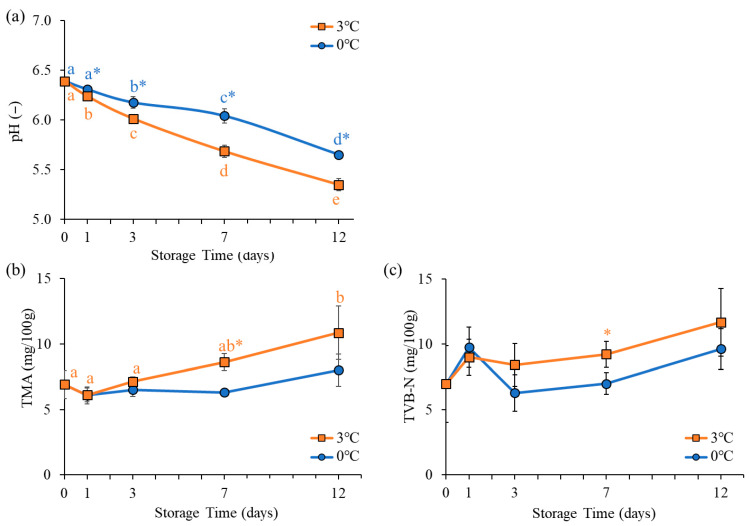
Time-dependent changes in (**a**) pH, (**b**) Trimethylamine (TMA), and (**c**) Total volatile basic nitrogen (TVB-N) in oyster meat during storage at different temperatures. Different markers show the mean values of different temperature conditions, while the bars indicate standard deviation at each storage time. Different lowercase letters indicate significant changes (*p* < 0.05) during storage time. * indicates significant variations in storage temperature (*p* < 0.05).

The results of pH, TMA, and TVB-N measurements in oyster meat are presented in Figure 4. pH is widely recognized as a primary physicochemical indicator of seafood freshness [31]. In the present study, pH values significantly decreased during storage under both temperature conditions. The decrease was more pronounced at 3 °C, where values remained significantly lower than those at 0 °C from day 1 onward. Cao et al. [25] also reported that oyster meat pH decreased during storage and that this decrease was reduced by low temperatures. In this study, among the VOCs, acetic acid and pentanoic acid increased significantly during storage (as described in Section 3.5). Furthermore, Tanimoto et al. [11] reported that the levels of non-VOCs, such as succinic acid and acetic acid, increased during refrigerated storage of shucked oyster meat. Additionally, Chiou et al. [18] reported that storage at 5 °C promoted the increase in succinic acid in clams than that of storage at 0 °C, suggesting that storage temperature may be involved in the production of substances that cause pH reduction.

TMA is a volatile compound produced from trimethylamine oxide (TMA-O) via bacterial and enzymatic activities, particularly under anaerobic conditions [31]. TVB-N represents the sum of volatile nitrogenous compounds, including TMA, dimethylamine, and ammonia, and is commonly used as an indicator of seafood spoilage. In the present study, a significant increase in TMA was observed only under 3 °C storage conditions. In contrast, TVB-N levels did not change significantly during storage at both storage temperatures. However, from day 3 onward, the mean TVB-N values were consistently lower at 0 °C than at 3 °C, with a statistically significant difference observed on day 7 of storage. The observed variation in TVB-N attributable to storage conditions may reflect an increase in TMA levels at 3 °C. An increase in temperature from 0 °C to 3 °C led to a significant elevation in TMA, a compound linked to the fishy odor characteristic of seafood [32]. This finding suggests that the storage temperature influences the flavor profile of oysters.

### 3.4. Sensory Evaluation and Odor Sensor Analysis

The results of the sensory evaluation and odor sensor measurements are presented in Table 3. The intensity of the odor and the spoilage odor of oyster meat increased significantly during storage at 3 °C, whereas no significant increase was observed at 0 °C. On day 12, the intensity of the odor was rated significantly lower at 0 °C than that at 3 °C. In the brine samples, odor intensity increased significantly under both storage conditions. Brine stored at 0 °C for 7 days demonstrated a significantly lower odor intensity than that stored at 3 °C, highlighting a temperature-dependent change in the brine. These significant distinctions were observed earlier than in the oyster meat.

Regarding odor palatability, oyster meat samples stored at 0 °C were evaluated as significantly more pleasant than those stored at 3 °C on day 12. These findings suggest that ice storage mitigates the progression of unpleasant odors in oyster meat. Moreover, this difference in odor emerged earlier in the brine than in the oyster meat.

Odor sensor measurements supported the sensory evaluation results. For oyster meat, sensor values were significantly lower on day 12 at 0 °C than that at 3 °C. In brine, significant differences were observed on day 3 and day 7, with lower values at 0 °C than 3 °C. The results of this instrumental analysis demonstrate consistency with the sensory evaluation findings, indicating that odor intensity and its changes can be monitored more accurately using these analytical methods. Collectively, these findings confirm that ice storage conditions effectively inhibit the development of unpleasant odors in oyster meat and brine.

### 3.5. Volatile Component Analysis

**Figure 5:**
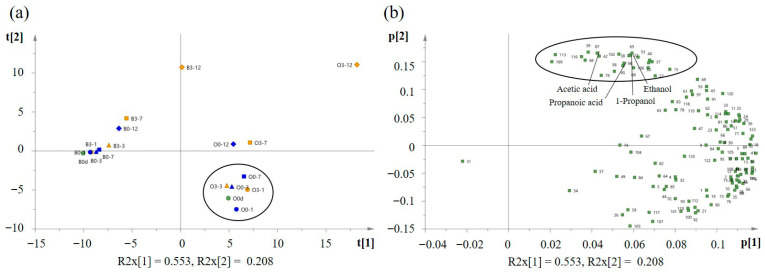
Principal component analysis plot by volatile organic compounds (VOCs) in oyster meat and brine during storage at different temperatures. (**a**) Score plot. O: oyster meat; B: brine. 0 °C: blue, 3 °C: orange. Day 0: circle (green color), Day 1: circle, Day 3: triangle, Day 7: square, Day 12: diamond. (**b**) Loading plot, the numbers indicate the peak numbers of VOCs, corresponds to Supplementary Tables S1 and S2.

In total, 125 VOCs were detected (Tables S1 and S2). Figure 5 presents a principal component analysis (PCA) plot based on the VOC data from all samples. The first principal component (PC1) accounted for 55.3% of the total variance, while the second principal component (PC2) explained 20.8%. The score plot (Figure 5a) showed that PC1 clearly distinguished the oyster meat from the brine. In the brine samples, the storage caused their plots to be shifted diagonally to the right. The sample from day 12 at 3 °C was positioned at the top right. The change in scores for the 0 °C storage samples was smaller compared to the 3 °C samples, and the sample from day 12 at 0 °C was positioned between the samples from days 3 and 7 at 3 °C. These results suggest that 0 °C storage inhibited the generation of VOCs in the brine compared to 3 °C storage.

For oyster meat, the sample from day 12 at 3 °C was positioned at the upper right, the plot for day 12 at 0 °C was close to that for day 7 at 3 °C. This is consistent with the change in VOC behavior reported by Kawabe et al. [19] for shelled oysters stored at different temperatures. In addition, this indicated that VOC production changes are also suppressed at 0 °C in oyster meat. These findings are similar to the results of the PCoA (Figure 3), suggesting that changes in bacterial composition and VOCs show comparable trends. However, the samples during early storage were almost identically positioned. Consequently, PCA was performed again using only oyster meat (Figure S1). As a result (Figure S1a), the samples shifted to the left owing to storage, confirming that this change was decreased by storage at 0 °C. It suggested that changes in oyster meat VOCs were suppressed even at the early stage of storage at lower temperatures.

The loading plot (Figure 5b) showed that short-chain fatty acids, 1-propanol, and ethanol were all located in the upper right of the loading plot. Detailed VOC analysis (Table S1) revealed that in the brine, the contents of 1-propanol and acetic acid at 3 °C showed a significantly higher value than that at 0 °C from day 1 of storage. Furthermore, ethanol levels significantly higher at 3 °C from day 3 of storage. According to previous reports, these compounds significantly increased in oyster meat and brine [12,19], suggesting that they likely contributed most significantly to the VOC changes on day 12 at 3 °C (Figure 5a). In addition, the production of pentanoic acid, propyl propanoate, ethyl propionate, and 2-butanone is suppressed at low temperatures. Esters are formed owing to the reaction of alcohols and acids produced by the degradation of lipids and proteins in oysters [33]. Differences in ester formation during storage at different temperatures may be related to the suppression of alcohol (1-propanol and ethanol) and acid (such as acetic acid) production during the early storage period. Ultimately, on day 12 of storage, 51 VOCs showed significantly lower values at 0 °C than at 3 °C, indicating the decreased production of many VOCs.

In the oyster meat (Figure S1b), similar to that in the brine, short-chain fatty acids 1-propanol and ethanol were confirmed to contribute to spoilage progression. Detailed analysis (Table S1) revealed that by the end of storage, 18 VOCs showed significantly lower values at 0 °C than at 3 °C, confirming that low-temperature storage also affected oyster meat VOC production levels. Notably, 1-propanol showed significantly higher values at 3 °C from day 1 of storage, consistent with the brine results. In contrast, short-chain fatty acids such as acetic acid and propanoic acid showed significant temperature-dependent differences only from day 7 of storage, suggesting a delayed onset of their differences compared to the brine. In deteriorated oysters, organic acids increase and produce a strong sour odor [34], suggesting that organic acid production is likely to be involved in generating unpleasant odors. Furthermore, the loading plot for oyster meat indicated that aldehydes were positioned farthest to the left, suggesting that they likely contributed to the VOC changes during storage. Thus, aldehyde formation during storage were suppressed at 0 °C. Aldehydes are associated with lipid decomposition in fish meat and have low odor thresholds [33,35,36]. Therefore, the suppression of these VOCs at 0 °C may have contributed to the suppression of unpleasant odors in oyster meat at 0 °C (Table 3).

### 3.6. Correlation Between Bacteria and VOCs

**Figure 6:**
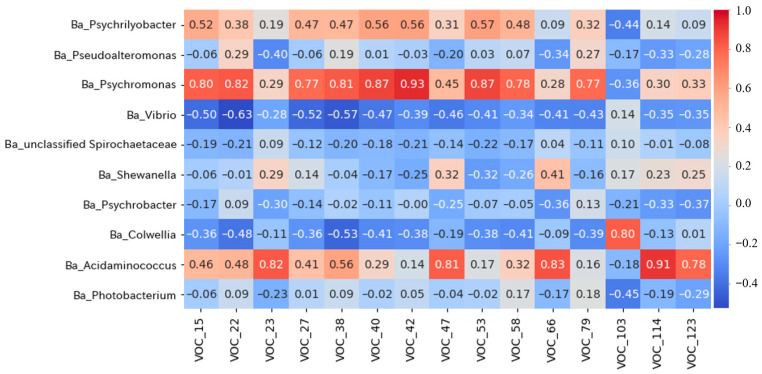
Heatmap of correlation coefficient between bacterial genera and volatile organic compounds (VOCs). The value following VOC on the horizontal axis indicates the peak numbers of VOCs, corresponds to Supplementary Tables S1 and S2. Correlation coefficients are indicated by the color scale on the figure.

Bacteria utilize nutrients, such as carbohydrates and proteins, contained in food for growth and proliferation, producing various metabolites [37,38]. VOCs are generated when bacteria break down nutrients into soluble, low-molecular-weight components; some of these VOCs cause unpleasant or off-odors [39]. Therefore, understanding the relationship between bacteria and VOCs is crucial for understanding the mechanisms of unpleasant odor generation. Therefore, a correlation analysis was performed between all VOCs and the top 15 dominant bacterial genera in the oyster meat and brine. The results for the top 15 VOCs showing a significant positive correlation (*p* < 0.05) are presented in Figure 6. The bacterial genera with significant correlations were mainly *Psychromonas* and *Psychrilyobacter*, which showed a high correlation with ethanol, 1-propanol, and esters such as ethyl acetate and ethyl propanoate. *Psychromonas* increases ethanol concentration during storage of packed blue mussels [40] and plays a key role in protein and lipid degradation [28]. Additionally, in mandarin fish, VOCs generated during fermentation and *Psychrilyobacter* and *Acidaminococcus* are highly correlated [41]. *Psychrilyobacter* is involved in protein degradation, and short-chain fatty acids (acetic acid, propionic acid, and butyric acid) are produced through protein and lipid metabolism [28]. Since bacterial species utilize the generated short-chain fatty acids to produce esters as metabolic intermediates [42], the high correlation between bacteria and esters reflects this process. Furthermore, the higher correlation observed for *Psychromonas* than for *Psychrilyobacter* is likely because *Psychromonas* is involved in degrading proteins and lipids, supporting the findings reported by Pelikan et al. [28]. In the present study, the high correlation of these bacteria with many VOCs may also support their involvement in odor generation. However, further detailed investigations are required to determine the relationship between bacteria and VOC generation.

## 4. Conclusions

This study investigated the effects of storage temperature on the quality of shucked oysters soaked in seawater, with a specific emphasis on quality indicators, sensory characteristics, and microbial communities. The results demonstrated that low-temperature storage (0 °C) suppressed changes in viable bacterial counts, bacterial communities, TVB-N, and odor-related compounds such as TMA and VOCs, compared to storage at 3 °C. Sensory evaluation and odor sensor measurements further confirmed that unpleasant odor development was significantly reduced under ice storage conditions. The dominance of specific bacterial genera was temperature-dependent, with certain spoilage-associated bacteria proliferating at higher temperatures, while these bacteria remained stable under ice-storage. These bacteria remained unchanged under ice-storage conditions. Changes in the VOC profiles of the oyster meat and brine were observed during storage at both temperatures. Storage at 0 °C delayed this VOC profile change compared to storage at 3 °C, and oyster meat aldehyde formation was particularly suppressed at 0 °C. Correlation analysis revealed that *Psychromonas* and *Psychrobacter* were positively correlated with several VOCs. These results indicate that even a slight temperature difference between 0 °C and 3 °C affects the quality of shucked oysters, highlighting the importance of storing them at lower temperatures with strict temperature control. Future research should investigate the effectiveness of colder storage conditions, such as super-chilled environments, in maintaining the quality of shucked oysters and explore more advanced storage techniques to further extend their shelf life. A limitation of this study is that the influence of the indigenous bacteria present in the seawater used for immersion was not fully elucidated. Future research should focus on evaluating the effects of sterilized brine on microbial dynamics and quality preservation to better understand the role of environmental microbiota in oyster spoilage.

## Figures and Tables

**Table 1:** Sensory evaluation score and criteria for quality assessment.

		Sensory Evaluation Score				
		1	2	3	4	5
Oyster	Odor intensity	very weak	weak	normal	strong	very strong
	Spoilage odor intensity	very weak	weak	normal	strong	very strong
	(object of reference)	(OY; day 0)				(OY; 3 °C, day 12)
	Odor palatability	very unpalatable	unpalatable	normal	palatable	very palatable
	(object of reference)					(OY; day 0)
Brine	Odor intensity	very weak	weak	normal	strong	very strong
	(object of reference)	(BR; day 0)				

The items in parentheses indicate the samples used as object of reference when evaluating for sensory evaluation. OY: oyster meat; BR: brine.

**Table 2:** Alpha diversity of the microbiome in oyster meat during storage at different temperatures.

Alpha Diversity	Storage Temperature	Oyster Meat			Brine		
		0 (Days)	3 (Days)	12 (Days)	0 (Days)	3 (Days)	12 (Days)
Good’s coverage	0 °C	1.0 ± 0.0	1.0 ± 0.0	1.0 ± 0.0	1.0 ± 0.0	1.0 ± 0.0	1.0 ± 0.0
	3 °C	1.0 ± 0.0	1.0 ± 0.0	1.0 ± 0.0	1.0 ± 0.0	1.0 ± 0.0	1.0 ± 0.0
ASV	0 °C	91.0 ± 20.5	74.0 ± 15.4	94.0 ± 24.1	153 ± 38.2	113 ± 13.7 *	95 ± 14 *
	3 °C	91.0 ± 20.5	82.7 ± 13.0	84.7 ± 15.3	153 ± 38.2 a	74 ± 8.1 b	81 ± 8 b
ACE	0 °C	91.4 ± 20.6	74.0 ± 15.4	94.7 ± 24.2	153 ± 38.6	114 ± 13.1 *	97 ± 14
	3 °C	91.4 ± 20.6	83.0 ± 12.5	85.7 ± 16.2	153 ± 38.6 a	74 ± 8.9 b	83 ± 12 b
Chao1	0 °C	91.3 ± 20.5	74.0 ± 15.4	94.7 ± 24.2	143 ± 48.5	104 ± 22.6 *	104 ± 8
	3 °C	91.3 ± 20.5	83.0 ± 12.5	85.7 ± 16.3	129 ± 65.3 a	77 ± 8.1 b	85 ± 16 b
Shannon	0 °C	3.9 ± 0.8	3.2 ± 1.0	4.1 ± 1.1	6.0 ± 0.2 a	5.0 ± 0.3 b*	4.6 ± 0.4 b
	3 °C	3.9 ± 0.8	3.4 ± 0.7	4.2 ± 0.4	6.0 ± 0.2 a	4.2 ± 0.2 b	4.4 ± 0.3 b
Simpson	0 °C	0.8 ± 0.1	0.8 ± 0.2	0.8 ± 0.1	1.0 ± 0.0	0.9 ± 0.0	0.9 ± 0.0
	3 °C	0.8 ± 0.1	0.8 ± 0.1	0.9 ± 0.0	1.0 ± 0.0	0.9 ± 0.0	0.9 ± 0.0

The data are presented as means ± standard deviation (*n* = 3). Different lowercase letters indicate significant changes (*p* < 0.05) during storage time. * indicates significant variations in storage temperature (*p* < 0.05).

**Table 3:** Sensory evaluation and odor sensor measurements in oyster meat and brine during storage at different temperatures.

	Samples	Evaluation Items	Storage Temperature	0 (Days)	1 (Days)	3 (Days)	7 (Days)	12 (Days)
Sensory score	Oyster meat	Odor intensity	0 °C	2.5 ± 1.4	2.1 ± 1.2	2.4 ± 1.2	2.4 ± 1.3	2.8 ± 1.1
			3 °C	2.5 ± 1.4 a	2.9 ± 1.1 ab*	2.6 ± 1.0 ab	2.4 ± 1.2 a	3.6 ± 1.2 b*
		Spoilage odor intensity	0 °C	2.5 ± 1.3	2.0 ± 1.1	2.4 ± 1.2	2.1 ± 1.2	2.8 ± 1.3
			3 °C	2.5 ± 1.3 a	2.5 ± 1.1 a	2.9 ± 1.1 ab	2.7 ± 1.2 a	3.6 ± 1.3 b*
		Odor palatability	0 °C	3.5 ± 1.3	3.7 ± 1.1	3.7 ± 1.1	3.7 ± 1.1	3.3 ± 1.2
			3 °C	3.5 ± 1.3 a	3.6 ± 1.0 a	3.0 ± 1.1 ab	3.3 ± 1.2 ab	2.5 ± 1.3 b*
	Brine	Odor intensity	0 °C	1.4 ± 0.8 a	2.4 ± 1.1 b	2.1 ± 1.0 b	2.5 ± 1.2 b	3.4 ± 1.1 c
			3 °C	1.4 ± 0.8 a	2.4 ± 1.2 b	2.3 ± 1.1 b	3.6 ± 1.2 c*	3.9 ± 1.1 c
Odor sensor	Oyster meat	Odor intensity	0 °C	16.8 ± 2.0	17.4 ± 2.6	18.4 ± 2.3	19.2 ± 3.1	22.0 ± 0.7
			3 °C	16.8 ± 2.0 a	16.9 ± 1.2 a	20.3 ± 2.1 ab	22.6 ± 1.6 bc	25.3 ± 0.8 c*
	Brine	Odor intensity	0 °C	0.4 ± 0.7 a	3.3 ± 1.7 ab	4.2 ± 0.9 b	12.0 ± 2.2 c	22.4 ± 0.8 d
			3 °C	0.4 ± 0.7 a	4.2 ± 0.9 b	14.0 ± 0.2 c*	22.1 ± 0.9 d*	24.9 ± 0.6 e

The data represent the means ± standard deviation. Different lowercase letters indicate significant changes (*p* < 0.05) during storage time. * indicates significant variations in storage temperature (*p* < 0.05).

## Data Availability

The original contributions presented in this study are included in the article/Supplementary Materials. Further inquiries can be directed to the corresponding author.

## Supplementary Materials

The following supporting information can be downloaded at https://www.mdpi.com/article/10.3390/foods15040603/s1: Figure S1: Principal component analysis plot by volatile organic compounds (VOCs) in oyster meat during storage at different temperature; Table S1: Volatile organic compounds in oyster meat during storage at different temperatures; Table S2: Volatile organic compounds in brine during storage at different temperatures.

## Abbreviations

The following abbreviations are used in this manuscript:

ANOVA	one-way analysis of variance
ASV	amplicon sequence variant
CFU	colony-forming units
GC-MS	gas chromatography–mass spectrometry
PC1	first principal component
PC2	second principal component
PCA	principal component analysis
PCoA	principal coordinate analysis
PCR	polymerase chain reaction
TMA	trimethylamine
TMA-O	trimethylamine oxide
TVB-N	total volatile basic nitrogen
VOCs	volatile organic compounds
