# Supplementary material for: Effects of Storage Temperature on the Microbial Flora, Odor, and Quality of Shucked Pacific Oysters Under Mimicked Commercial Shipping Conditions
Source: Foods. 2026 Feb 7;15(4):603. doi: 10.3390/foods15040603 (PMC12939646; doi:10.3390/foods15040603)
Supplement: Supplementary file 1 [file foods-15-00603-s001.zip › FigureS1.pptx]

## Slide 1
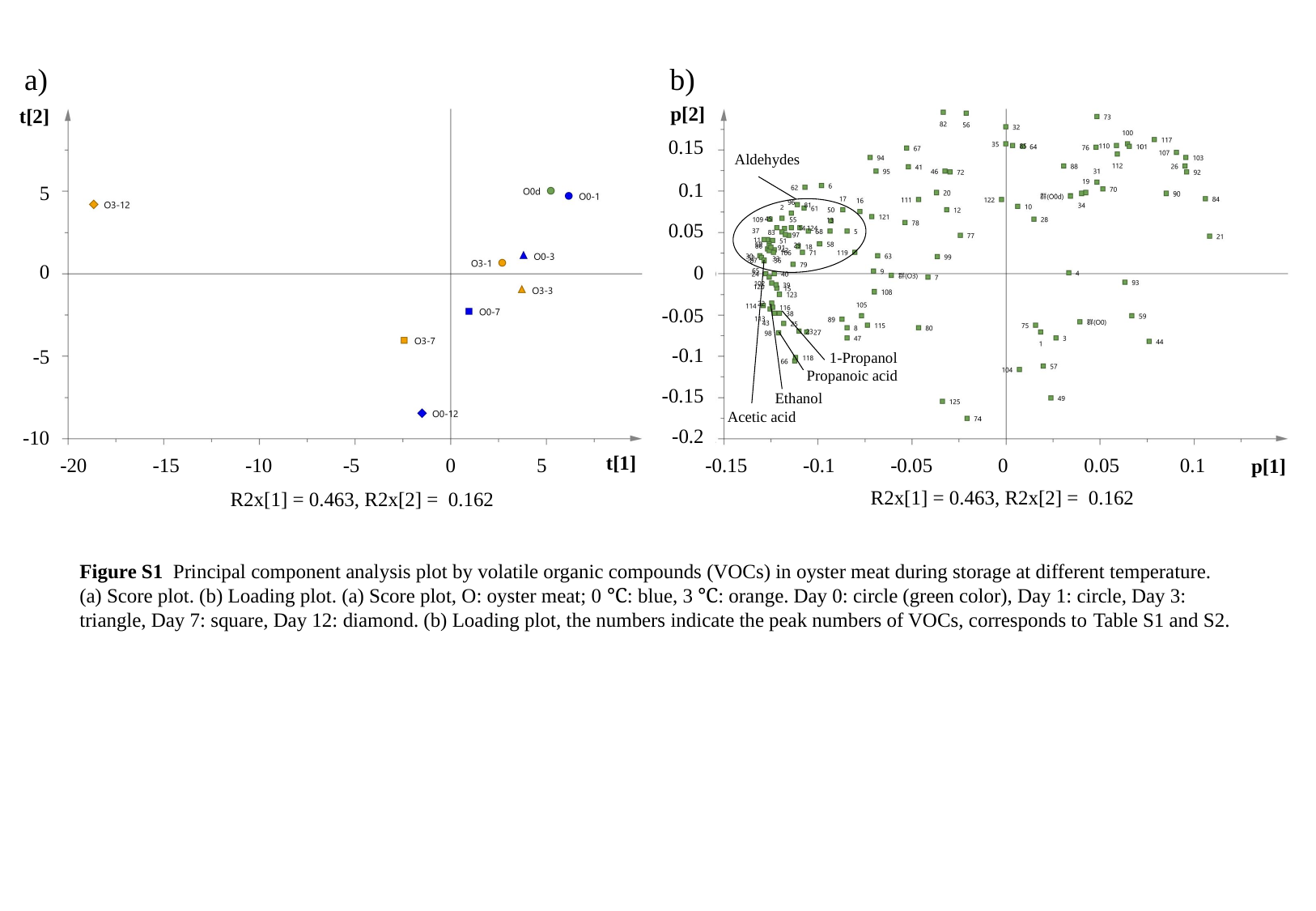

a) b)
p[2]
1-Propanol
Propanoic acid
Ethanol
Acetic acid
t[2]
0.15
0.1
0.05
0
-0.05
-0.1
-0.15
-0.2
Aldehydes
5
0
-5
-10
t[1]
-20 -15 -10 -5 0 5
-0.15 -0.1 -0.05 0 0.05 0.1
p[1]
R2x[1] = 0.463, R2x[2] = 0.162
R2x[1] = 0.463, R2x[2] = 0.162
Figure S1 Principal component analysis plot by volatile organic compounds (VOCs) in oyster meat during storage at different temperature.
(a) Score plot. (b) Loading plot. (a) Score plot, O: oyster meat; 0 ℃: blue, 3 ℃: orange. Day 0: circle (green color), Day 1: circle, Day 3: triangle, Day 7: square, Day 12: diamond. (b) Loading plot, the numbers indicate the peak numbers of VOCs, corresponds to Table S1 and S2.
